# Experiences of refugees and asylum seekers in general practice: a qualitative study

**DOI:** 10.1186/1471-2296-8-48

**Published:** 2007-08-21

**Authors:** Ravi Bhatia, Paul Wallace

**Affiliations:** 1 Department of Primary Care and Population Sciences, Royal Free and University College Medical School, Holborn Union Building, Archway Campus, Highgate Hill, London, N19 5LW, UK

## Abstract

**Background:**

There has been much debate regarding the refugee health situation in the UK. However most of the existing literature fails to take account of the opinions of refugees themselves. This study was established to determine the views of asylum seekers and refugees on their overall experiences in primary care and to suggest improvements to their care.

**Methods:**

Qualitative study of adult asylum seekers and refugees who had entered the UK in the last 10 years. The study was set in Barnet Refugee Walk in Service, London. 11 Semi structured interviews were conducted and analysed using framework analysis.

**Results:**

Access to GPs may be more difficult for failed asylum seekers and those without support from refugee agencies or family. There may be concerns amongst some in the refugee community regarding the access to and confidentiality of professional interpreters. Most participants stated their preference for GPs who offered advice rather than prescriptions. The stigma associated with refugee status in the UK may have led to some refugees altering their help seeking behaviour.

**Conclusion:**

The problem of poor access for those with inadequate support may be improved by better education and support for GPs in how to provide for refugees. Primary Care Trusts could also supply information to newly arrived refugees on how to access services. GPs should be aware that, in some situations, professional interpreters may not always be desired and that instead, it may be advisable to reach a consensus as to who should be used as an interpreter. A better doctor-patient experience resulting from improvements in access and communication may help to reduce the stigma associated with refugee status and lead to more appropriate help seeking behaviour. Given the small nature of our investigation, larger studies need to be conducted to confirm and to quantify these results.

## Background

The increasing refugee [throughout this report, the term "refugee" is used to denote "refugee and asylum seeker"] population in the UK has led to increased research and debate about their health and social needs [1]. Most studies have been based on health professionals' views of how refugees should be managed and the problems encountered by these professionals when dealing with them. They have exposed how healthcare for refugees is patchy and often inappropriate [2] with inequalities in relation to access adversely affecting refugee health [3-5]. Refugees are perceived by general practitioners (GPs) to have multiple needs that are difficult to fulfil and as a result some are even refused registration [6,7]. To help the situation non-governmental organisations have set up clinics in certain areas for vulnerable migrants who have difficulty accessing primary care, but there are concerns over their ability to provide continuity to care, refer to secondary care and their use of local resources such as GPs and nurses [8,9].

Government legislation has often hindered efforts to improve the refugee health situation. Denying failed asylum seekers access to free secondary care and proposals to extend this to non-urgent primary care have been labelled unethical and put greater pressure on already overburdened A&E services [10,11]. Active re-location of refugees away from points of arrival in South East England has been associated with increased rates of temporary GP registration [3] and therefore removes any financial incentive for GPs to perform immunisation and cervical smear tests in this population. Consequently, this puts refugees at increased risk of disease [12-14]. Re-location also results in refugees moving to areas where appropriate services have not been developed, potentially leading to reduced access to services such as interpreters or refugee community groups [15-17].

Language problems prevent GPs understanding patients' needs, leading to decreased symptom reporting and fewer appropriate referrals to secondary care [18-20]. Interpreters are vital in helping to overcome both bi-lingual and inter-cultural communication. Increased cultural awareness and sensitivity has been shown to facilitate communication, management and compliance in cross cultural consultations [21-23]. Cultural awareness is especially important when caring for refugee populations due to their limited English, differing explanatory models and differing models of distress which all lead to difficulty in diagnosis and engaging in treatments such as psychological therapies [24-27]. Without interpreters refugees may also acquire inappropriate prescribed medications because doctors have been unable to take an adequate history [6]. However, organising professional interpreting is difficult, especially in emergency consultations or in areas where there are small numbers of different ethnic groups and children are often used instead [28]. There are many ethical concerns over the use of lay interpreters, especially children and many GPs and academics have come to a consensus that the use of professional interpreters should be the gold standard in bilingual consultations [19].

Despite the growing emphasis on patient and public involvement in healthcare provision, there are few studies using data from refugees themselves. Those studies which have used data from refugees, have usually either been conducted outside the UK, restricted to a specific ethnic group or to a particular topic such as mental health or inter cultural communication [5,19,21,29]. This study was designed to determine the views of refugees about their overall experiences of general practice. We also sought to identify what they saw as problems when dealing with general practices and practitioners and how they would improve their experiences in primary care.

## Methods

Due to the exploratory nature of the research question and the fact that resources were not available to validate questionnaires into multiple languages, a qualitative study design was used. We carried out semi-structured interviews at the Barnet Refugee Service (BRS) drop-in, a Monday morning walk in service in North London designed to support refugees with difficulty accessing health, housing, employment, interpreting and legal services [30]. Although BRS had no quantitative information of the demographics of those using the service, they informed the researchers that refugees of varying ages and ethnic backgrounds visited the drop in service. Researchers were informed that refugees from Somalia and Farsi speakers from Afghanistan were more likely to visit the drop in due to their high numbers in the local area. All refugees aged 18 or over visiting the drop in service between February and March 2006 were invited to participate in the study by a member of staff at the centre so long as they could speak English or an interpreter was available on that day to translate an interview. Although the exact response rate was not recorded, approximately 15–20 refugees were invited to participate and most refugees agreed to be interviewed. The most common reasons for refusing to participate were a lack of time or anxiety regarding confidentiality due to the fact that the interviews were to be recorded and that the subject matter of the interviews related to healthcare. Because of the the concerns held over confidentiality of the data, we were unable to collect the demographics of those that refused to be interviewed therefore unable to compare this to those that agreed to participate.

Those who consented underwent a screening interview to determine eligibility for the study. Refugees were eligible for inclusion provided they had been resident in the UK for less than 10 years and were either applying for asylum, had been granted refugee status, indefinite or exceptional leave to remain in the UK, or had been refused asylum. Written consent was sought from all eligible subjects, but verbal consent was accepted from those unable to read or complete the form. The semi structured interviews were 45 minutes long and conducted with assistance from an interpreter if needed. Topics included in the interviews were derived from a review of the relevant research literature. These included the process of finding, registering and getting to see a GP, the respondents' experiences of the GP consultation and their views about ways to improve refugees' experiences in primary care [See Additional File 1]. However a fluid interview style was used, so as not to impose the researchers' agenda on the interview. RB conducted all the interviews, recorded and transcribed the interviews verbatim and analysed the transcripts. There is an obvious disadvantage in using interpreters in qualitative studies as the data is modified before analysis. Although the bias can be reduced by back translating transcripts, this was not done, again due to lack of resources. However, only three respondents (6, 8 and 9) required an interpreter.

Analysis of the interviews was undertaken using the framework approach outlined by Pope et al and Ritchie and Spencer [31,32]. This method was chosen due to time restraints and because this method is often used in health policy research contexts [33]. The method involved familiarisation with the data (reading and re-reading the data) and identifying the coding framework (key themes which emerged both from the aims of the study as well as from the actual transcripts). The coding framework was then indexed (marking quotations which belonged to either one theme or another) and then charted (where the quotations were rearranged under each theme). This was repeated for each theme that emerged. The final process included mapping and interpreting the data, which involved drawing similar themes together and trying to explain why themes emerged. Plans to put the themes that emerged from the interviews to a focus group in order to improve reliability of the study had to be abandoned due to the lack of multi-lingual interpreters.

Ethical and research governance approval were obtained from Barnet Local Research and Ethics Committee and from the North Central London Research Consortium respectively in January 2006.

## Results

Interviews were successfully completed with a total of 11 respondents (Table 1). We identified three themes from the analysis of the interviews: difficulties encountered by refugees in the healthcare system, the impact of these difficulties and how refugees would like to improve their experiences in the UK healthcare system.

**Table 1:** Attributes of participants

	**Age**	**Sex**	**Country of origin**	**First language**	**Asylum status**	**Benefits received**	**Housing status**	**Employment status**
**1**	22	F	Somalia	Somali	Failed & appealing	None	Lives with friends	Not allowed to work
**2**	50	F	Nigeria	English	Failed	None	Lives with friends	Not allowed to work
**3**	37	M	Sri Lanka	Sinhalese	Applying	None	Awaiting council housing	Not allowed to work
**4**	65	M	Eritrea	Tigrinya	Failed & appealing	None	Council housing removed when refused	Unemployed (over age of retirement)
**5**	35	F	Azerbaijan	Russian	Applying	None	Lives with friends	Not allowed to work
**6**	35	F	Afghanistan	Farsi	Refugee	Income support	Council housing	Unemployed (medically unfit)
**7**	34	F	Uganda	Soga	Failed & appealing	Child benefit	Council housing	Not allowed to work
**8**	36	F	Afghanistan	Farsi	Applying	Income support	Council housing	Unemployed (medically unfit)
**9**	24	F	Afghanistan	Farsi	Failed & appealing	None	Lives with sister in council house	Not allowed to work
**10**	47	F	Somalia	Somali	Indefinite leave to remain	Income support	Council housing	Unemployed (medically unfit)
**11**	?	F	Nigeria	English	Exceptional leave to remain	Income support	Council housing	Unemployed but volunteering

## Difficulties encountered by refugees in the healthcare system: (Table 2)

**Table 2:** (Number in brackets denotes interview number)

**Difficulties registering with a GP:**
*"The GP... says that I should go and get my letter from the home office before they should treat me, so I don't have GP" (2)*

**Difficulties making appointments due to language:**
*"We didn't have telephone. But... reception say you don't come, you have to call. We can't speak on phone. If you see on the face it's easier" (5)*

**Refused access to GP when refused asylum-**
*"I've received a letter saying that since you have been refused that we can not help you any more" (9)*

**Refusal of interpreter by the GP-**
*"They said that we provide you interpreter in hospital but in GP we cannot provide you interpreter" (9)*

**Poor continuity of care-**
*"Wood Green one is very nice... My Hendon [GP] was very, very, very good... [Then] I moved to Cranbourne; after I move again, I come [back to] Hendon; I come back my old GP." (10)*

**Experience of not having one doctor even at one practice-**
*"I didn't know the real GP because they used to tell me this is your GP sometimes they find another person, sometimes you see another one (7)*

**Perceived stigma and discrimination-**
*"I heard [a worker at a hospital] talking over the phone... [He said], " [I'm] playing a game and [I'm] just using story to claim asylum ... as [a] mentally ill [patient]."(3)*

*"Being an asylum seeker ... you feel people look at you as if you're not a human being [but] you're something different" (11)*

*"As soon as she [nurse] realised we were refugees she started not listening to us and treated us differently" (9)*

There were difficulties encountered in many aspects of healthcare. The two problems people cited when trying to access GPs were firstly problems locating practices and then language difficulties once arriving at them. This led to problems both registering with a practice and also making an appointment. Some respondents also told of how they initially found it easy to register, but were subsequently denied healthcare after their asylum claim was refused.

Once in the consulting room, there was a general consensus that difficulties in language constituted a significant barrier to effective healthcare. Although most respondents reported that they could ask for a professional interpreter when they liked, one said her request had been refused outright by her GP. Some respondents mentioned that there were times when appointments had to be re-booked because the interpreter failed to attend, and others mentioned that for emergency appointments, it was frequently not possible to book a professional interpreter.

A number of respondents told how they had moved from one GP to another, because they had been told to move by the National Asylum Support Service or by their local council. Even when they had settled in one practice, some told how they were not given one GP and consequently this meant they were unable to build up a relationship with a doctor.

Several respondents voiced their concerns at the prevailing hostile attitudes in the UK towards refugees. This did not lead to outright hostility but to a perceived atmosphere in the healthcare system where refugees were seen as unwanted and as a burden on resources.

## Impact of the difficulties faced in the healthcare system on refugees: (Table 3)

**Table 3:** 

**Easier registration with support-**
*"First time my English was bad, they didn't listen us because they said they no understand me and no translator. Have to find friend to bring me and explain what's problem". (5)*

**Shopping around to find a GP-**
*"My husband's GP say they can't register me because I'm an asylum seeker...I had to move ... [In the new GP] they didn't ask me questions like the other one" (7)*

**Unreliability of family as interpreters in emergency consultations-**
*"My sister has got the children but she can not come all the time" (9)*

***Views on use of male interpreters by women***
*"It doesn't make any difference to me."(9)*

**Use of family as interpreters-**
*"It's my daughter...my personal – I don't have secrets from my children... [they] know everything..." (10)*

**Cultural influences on views on interpreters-**
*"I don't like to talk to her [interpreter] because you know we fight in Somalia. ... That clan and that clan.... But I don't know who this lady is so I don't like to say that" (1)*

**Use of dictionaries as an aid to communication-**
*"People no understand Italian, but I buy dictionary, Somali and English. If I talk someone, I look in dictionary and after I speak a bit" (10)*

**Change in help seeking behaviour-**
*"Sometimes I feel they'll be fed up with me, especially a foreigner you know... [If] I don't like this medication, it's like I'm bothering them or so, so I don't go back to tell the doctor." (11)*

All of the participants stated that friends, family and support agencies were their main source of information regarding the location of their nearest GP and the documentation required for registration. It became apparent that *only *those who were accompanied by a friend, relative or refugee agency staff member experienced a trouble-free registration process. The variability of ease of registration with different practices, led to refugees "shopping around" for a suitable practice. Those who had found it hardest to register also had greatest difficulty in securing an appointment.

Many participants gave their experiences of how they overcame the language barrier with their GP. Because of some difficulty accessing professional interpreters, some turned to using friends and family; however it was noted that lay interpreters could be just as difficult to access in emergencies. Women did not seem troubled by gender discordant professional interpreters whether in emergency situations or not. A few felt that it was inappropriate to use children as interpreters, but others much preferred using their friends and family and were not troubled by using children as interpreters, even when talking about personal issues. Those respondents who much preferred the use of family and friends as interpreters voiced strong concerns over how much they could trust professional interpreters, stating that they did not talk openly to the doctor because they did not trust the interpreter. These respondents were both from Somalia, and cited inter-communal violence in their country of origin as a reason. A number of respondents mentioned that they were sometimes able to obtain the same interpreter each time they visited the GP but two respondents suggested that in the absence of an interpreter, bilingual dictionaries could be used, either before or during a consultation as an aid to communication.

Due to the stigma of being classed as a refugee, participants started to question how often they could reasonably access healthcare services with some stating that they were afraid to go to the doctor for fear of being thought of as using up too many resources.

## How refugees would improve their experiences in the UK healthcare system: (Table 4)

**Table 4:** 

**Desire for easier access to healthcare-**
*"It's unfair, because they should make medical care free for anybody who comes into the country whether they have papers or no papers" (2)*

**Irritation at lack of continuity of care-**
*I don't wanna move, move, move, move... but it's happened like that. ...The new GP no understand my problem (10)*

**Preference for the use of the same interpreter in each consultation-**
*"I have a connection with her and that makes me feel safer to have only one" (6)*

**How to change attitudes-**
*"The government should take time to enlighten people about why people come to this country... The reason is very important. Because nobody wakes up one day and says I'm fed up of living in my own country" (11)*

**Trying to fight discrimination with limited English-**
*When I try and complain ... [the receptionist] said go and complain and she give it me HSN [NHS] telephone number ... it's my English, of course nobody is gonna be listen my complaint. ... I was upset and I went home... [that was the] end [of] my complaint."(5)*

**Unease at doctors who fob off concerns-**
*"They don't really accept it, they just reject it. Without just saying something they say no it's not a problem, it's not like that." (4)*

**Preference for doctors that listen-**
*"He listens to me and he tries to find out the background of my illness. ... He tried to find out whether the illness was because the situation I found myself in." (11)*

**Preference for advice over medication-**
*"When ... doctor gives you advice about the small things ... you feel like he care. But here it's more Paracetemol, for ear or throat, which is not right." (5)*

Participants, especially failed asylum seekers desired easier access to GPs but they also wished for some continuity of care both with the GP and with any professional interpreter used. This allowed them to gain trust in the confidentiality of their interpreter as well as build up a relationship with the GP.

With regards to the perceived stigma in the health service towards refugees, respondents expressed that changes in attitude need to be made in society as a whole, rather than just within the healthcare sector. They wanted the population understand the reason why they had come to this country, rather than to assume that they were coming to use the free healthcare system. Respondents felt the discrimination needed to be dealt with in order to make them feel comfortable in the healthcare system, although when one participant tried to complain about discrimination herself, her limited English did not help.

Maybe it was because of linguistic difficulties, the lack of continuity of care and the perceived stigma of being a refugee that a number of participants felt their concerns had not been taken seriously by the GP and hence expressed negative views about their care. Others believed that these difficulties meant that GPs tended to rely heavily on medication and failed just to listen to them or provide appropriate advice. Most respondents explicitly stated that they valued doctors that listened to them, and almost all mentioned this quality when describing a good experience with a doctor. They also preferred doctors who were less reliant on handing out medication and more inclined to give advice instead. The use of traditional medicine was preferred for minor illnesses and was often taken when Western medication was thought to be ineffective or sometimes even concurrently with Western Medicine. In general, participants viewed traditional medicine as producing fewer side effects than Western medication.

## Discussion

### Corroboration with the literature and implication for future practice

A number of studies have shown that access to healthcare is difficult for refugees and that varying attitudes towards refugee health issues amongst doctors has been shown to substantially contribute to the difficulties experienced by refugees in gaining access to healthcare[3-5,15]. This may explain our reports of mixed access in different GPs which led patients to "shop around" for a GP willing to register them. GPs desire extra support to cater for the needs of refugees [33] and if it may be valuable for Primary Care Trusts (PCTs) to provide education and training, information and regular updates to GPs on the healthcare entitlements of the refugee population so that there is more uniformity in access.

It was expected that failed asylum seekers would have most difficulty accessing healthcare. Our study suggests that immigrants who are applying for asylum for the first time and who have no support in accessing healthcare may have an equally problematic experience. Therefore, it may be useful for all newly arrived asylum seekers to be given information regarding the location of their nearest GP and the documentation required to register or to be directed to local refugee agencies who can help with both the registration process and booking an appointment.

The reality of obtaining professional interpreters is difficult to achieve and those who had access to interpreters cited practical difficulties with interpreters not attending or arriving late, which again supports conclusions already in the literature [28]. Our study suggests that concerns over confidentiality of professional interpreters may be amplified where there are inter-communal tensions in the country of origin although was too small to conclusively conclude this. The phenomenon has been reported elsewhere [34] but is under-researched in terms of how quantifiable concerns are or which ethnic group may have the most reservations. Should this finding be confirmed in larger studies, it may be wiser for GPs to come to an agreement with their patient regarding who is used to translate, instead of assuming that a professional interpreter would be desired.

Those refugees that have a preference for interpreters seem to prefer using the same one each time they visit their GP and this approach may be useful to implement in practice to ally concerns over confidentiality. The use of bi-lingual dictionaries as an aid to communication both before and during the consultation is another interesting topic for future research.

Refugees voiced a strong preference for doctors that listened to their concerns rather than prescribe medication. This would be expected given this is found to some extent in non refugee patients also [35]. The preference for greater continuity of care, although expressed by non refugee patients too [36], has greater significance however, because this finding may be directly due to the impact of the government's forced dispersal policy. Although the extent of the policy on refugee health needs to be evaluated further, this study suggests that refugees feel that constant relocation impacts negatively on their health.

Social exclusion, poverty, unemployment, poor accommodation and language skills cause some refugees to feel stigmatised [5]. A poor experience of primary care, in part explained by a tendency for doctors to view refugees as unwanted and a burden on the National Health Service (NHS) [6,7,37] may also help enforce this stigma. What is more worrying is that such attitudes may interfere with refugees' willingness to seek healthcare. Again this is hard to confirm decisively in our limited study, but given its grave implications, we suggest that further research is conducted in order to determine how widespread this occurrence may be and how refugee help seeking behaviour may be modified should this be the case.

This study provides an outline of the experience faced by refugees attempting to access healthcare in one localised setting in London, UK. Despite this, many studies looking into experiences of refugees in other developed countries have shown that they face similar problems as uncovered in our study e.g. the marginalization of refugees and consequent stigma has been described in studies from Canada [38] and studies from America and Switzerland have stressed the importance of overcoming linguistic and cultural barriers[19,21]. This study is too limited to compare our results with studies from abroad. We suggest however, that given the similar problems of language and stigma, some of our findings e.g. what refugees desire in order to overcome these problems may be applicable in other developed countries although additional research is needed to confirm this.

The role of cultural issues such as illness beliefs affecting access to and the use of GPs were not explored in great detail in our study due to the fact that participants did not share a common cultural background and we therefore expected extreme diversity in possible responses. Some of our findings relating to the impact of culture on refugee healthcare are supported by previous studies, e.g. the use of Western medicine alongside traditional medication [5]. However, other findings, e.g. that women were not troubled by gender discordant interpreters are in sharp contrast with earlier results [21]. These erratic corroborations with previous literature may be due to our heterogeneous and small sample size and it is probably more useful to study the impact of culture on refugee healthcare in more detail when studies are concentrated on one ethnic population only.

### Strengths and weaknesses

The original aims of the study to allow refugees themselves to give their experience of primary care and voice their suggestions for improvements were met. By recruiting participants at the refugee centre rather than in a general practice, we were able to include asylum seekers whose claim had failed and were thus ineligible for GP registration. Given the fact that larger numbers of Somali and Afghanis were expected to use the drop in and five out of eleven participants were from these countries, we presume that the range of participants generally reflected the broad demographics of those who used the drop in service. This was not quantitatively tested however due to the lack of exact data about users of the service before the study was conducted.

The study was small, with only 11 respondents (most of whom were female) and was conducted in only one setting. Because of resource constraints, there was no independent translating of interviews or coding of the data, and because it was not possible to conduct the focus group, there was no triangulation of the data. We were unable to identify appropriate validated questionnaires in multiple languages, and so no quantitative data could be obtained to support the findings. It is therefore not appropriate to make generalisations from such a small and localised qualitative study and the results have been discussed with this in mind. Nevertheless, it is not in the nature of qualitative research to produce generalisable results but to generate hypotheses which can then be tested in larger quantitative studies. Many of our conclusions are consistent with those found in a larger qualitative study with related aims conducted in the same location [39]. However, we acknowledge that those conclusions which have not been reported extensively elsewhere (e.g. the worse access to GPs for those refugees without support, concerns over confidentiality of professional interpreters, the impact of the lack of continuity of care and the affect that the stigma is having on refugee help seeking behaviour) need to be interpreted with caution in the light of the limitations of the study and merit further research in larger studies.

## Conclusion

This study found that those refugees without support from friends, family and refugee agencies may have the most difficulty accessing GPs because of language difficulties and lack of knowledge regarding documentation required. This may be improved by local PCTs working with refugee agencies and the Home Office to provide information to GPs regarding the healthcare entitlements of refugees and by providing refugees assistance when they first arrive in a particular area.

Some participants voiced frustration at the practicalities of using professional interpreters whilst others stated that they felt that they were not able to trust that professional interpreters were confidential. Further research needs to quantify the level of mistrust towards interpreters. Nonetheless it would be good practice for GPs to come to a consensus with refugees over who is used to translate and not to assume that professional interpreters are always desired. Refugees seem to prefer the use of the same interpreter with each consultation, and it may be useful to implement this where possible.

Several participants mentioned the stigma attached to being a refugee in the NHS and how this made them reduce the number of times they accessed GPs for fear of being viewed as a burden on resources. The extent of this reduction in help seeking behaviour needs to quantified, but improving access to practices and ensuring adequate communication in the consultation may help the situation.

This was a small, qualitative study carried out in a single location focussing on a particularly topical subject which confirmed findings from previous literature. It presented a number of additional conclusions, but was too small to decisively confirm them. Some of these merit further investigation in larger studies in multiple settings with quantitative measures to determine how generalised they hold in the refugee population as a whole in the UK and to draw conclusions which will assist with future healthcare delivery for refugees.

## Competing interests

The author(s) declare that they have no competing interests.

## Authors' contributions

RB had the conceived of the study, participated in its design and coordination and drafted the manuscript. PW supported RB in all aspects of the study. All authors read and approved the final manuscript.

## Pre-publication history

The pre-publication history for this paper can be accessed here:



## Supplementary Material

Additional file 1 Interview guide. Questions used in the interviews. Click here for file
