# Supplementary material for: Experiences of refugees and asylum seekers in general practice: a qualitative study
Source: BMC Fam Pract. 2007 Aug 21;8:48. doi: 10.1186/1471-2296-8-48 (PMC2001193; doi:10.1186/1471-2296-8-48)
Supplement: Additional file 1 — Interview guide. Questions used in the interviews. [file 1471-2296-8-48-S1.doc]

***Interview Guide***

Tell me about yourself where you came from, asylum status etc?

**i)** Tell me about your experiences trying to find a doctor when you first came to the country?

How easy was it to register with a GP?

What health advice were you given when you entered this country?

What were your overall experiences of the healthcare system when you came to this country?

How easy is it to try to see a doctor now?

How are you treated by receptionists?

**ii)** Tell me about a good experience that you have had with a GP?

Tell me about a bad experience that you’ve had with a GP?

How well do you think the doctor listens and responds to your needs and worries?

What kinds of things does your doctor do to make you believe that he understands your needs?

What kinds of things does a doctor do to make you believe that he doesn’t understand your needs?

How does the doctor treat you when he has to examine you?

**What are your thoughts on being treated by a doctor of the opposite sex?**

What are your feelings towards Western medicine?

**Do you ever use traditional medicine, and if so what for?**

What are your experiences with using professional interpreters with a doctor?

**Would you mind an interpreter of the opposite sex?**

How confidential do you think interpreters are?

What are your thoughts about using family members as interpreters?

Have you ever been denied healthcare?

How difficult is it to pay for any medications?

**iii)** Overall, what are your views on the healthcare system in this country?

**What were your expectations of the healthcare system and have they lived up to it?**

What do you see as the main problems when it comes to dealing with doctors?

What would you like to see improved about the healthcare system?

**What are the differences between the healthcare in your country of origin and this country and which do you prefer and why?**

What kind of level of healthcare do you think you receive compared to the rest of the population?

**If not already discussed:**

**AGE**

**ASYLUM STATUS**

**WORK**

**BENEFIT**

**HOUSING**

**COUNTRY OF ORIGIN**

**LANGUAGES SPOKEN**

**Questions in bold were added during the data collection phase**
